# Pre-PCI SGLT2 inhibitors and contrast-induced nephropathy in acute myocardial infarction: A meta-analysis

**DOI:** 10.1097/MD.0000000000044215

**Published:** 2026-05-12

**Authors:** Yue Li, Wanyao Zhang, Rui Li

**Affiliations:** a Department of Cardiology, The People’s Hospital of Rongchang District, Chongqing, China; b Department of Oncology, The People’s Hospital of Rongchang District, Chongqing, China.

**Keywords:** 2 inhibitors, acute myocardial infarction, contrast, glucose cotransporter, induced nephropathy, percutaneous coronary intervention, sodium

## Abstract

**Background:**

Contrast-induced nephropathy (CIN), a common complication of percutaneous coronary intervention (PCI), adversely affects clinical outcomes by extending hospital stays and increasing healthcare costs. Importantly, CIN is linked to poor prognosis in acute myocardial infarction (AMI) patients. This study evaluated the preventive effect of pre-procedural sodium-glucose cotransporter-2 (SGLT2) inhibitors administration on CIN incidence in AMI patients who received PCI.

**Methods:**

A systematic search of PubMed, Web of science, and the Cochrane Library was performed for studies published up to December 12, 2024. Observational studies and clinical trials investigating pre-procedural SGLT2 inhibitors use in PCI-treated AMI patients were included. Following PRISMA guidelines, 2 researchers independently screened the literature, extracted the data, and assessed the bias risk. Data synthesis utilized Review Manager 5.3 with a random-effects model to address heterogeneity. The primary outcomes included CIN incidence (95% confidence intervals (CI)). The secondary outcomes included all-cause mortality, major adverse cardiovascular events (MACE), recurrent myocardial infarction, and heart failure (HF) readmission, which were analyzed via risk ratios(RR) and I² statistics.

**Results:**

Five studies involving 3301 patients (SGLT2 inhibitors group: 665; control: 2636) were analyzed. Compared with the control group, the SGLT2 inhibitors group demonstrated significantly lower risks of CIN (RR: 0.55, 95% CI: 0.41–0.73, *P* < .0001), all-cause mortality (RR: 0.49, 95% CI: 0.29–0.81, *P* = .005), MACE (RR: 0.33, 95% CI: 0.17–0.65, *P* = .01), and HF readmission (RR: 0.30, 95% CI: 0.16–0.56, *P* = .0001). No significant difference was observed in the recurrent myocardial infarction rates (RR: 0.88, 95% CI: 0.38–2.06, *P* = .77).

**Conclusion:**

Pre-procedural SGLT2 inhibitors use significantly reduces CIN incidence, mortality, MACE, and HF readmission in PCI-treated AMI patients, suggesting potential cardiorenal protective benefits.

## 1. Introduction

Acute myocardial infarction (AMI), including ST-segment elevation myocardial infarction and non-ST-segment elevation myocardial infarction, remains a leading cause of death and disability worldwide. AMI is characterized by the abrupt cessation of coronary blood flow, resulting in myocardial ischemia, injury, and necrosis.^[1–3]^ Timely percutaneous coronary intervention (PCI) can restore coronary blood flow and improve survival rates, making it a critical intervention for enhancing patient outcomes.^[4]^ However, PCI may be complicated by CIN, a condition that not only prolongs hospital stays and increases medical costs but is also strongly associated with adverse long-term outcomes.^[5]^ Therefore, effective prevention and reduction of CIN are urgent priorities in cardiovascular medicine.

The high prevalence of comorbidities such as diabetes and chronic kidney disease (CKD) in AMI patients further exacerbates the risk of CIN, highlighting the need for effective preventive measures.^[6]^ Sodium-glucose cotransporter-2 (SGLT2) inhibitors are a novel class of glucose-lowering agents used to treat type 2 diabetes mellitus (T2DM). These agents work by targeting the proximal tubules of the kidneys, inhibiting sodium-glucose cotransporters, reducing glucose reabsorption, and promoting glucose excretion, thereby lowering blood glucose levels.^[7,8]^ In addition, SGLT2 inhibitors confer broad cardiorenal protective effects through multiple interrelated mechanisms.^[9]^ First, they promote natriuresis and osmotic diuresis by inhibiting sodium and glucose reabsorption in the proximal tubules, thereby reducing blood volume, blood pressure, and cardiac preload. These effects contribute to improved hemodynamic stability and symptom relief in AMI patients with heart failure (HF).^[10]^ Second, SGLT2 inhibitors attenuate oxidative stress and inflammation – two central mechanisms underlying contrast-induced nephropathy (CIN) and myocardial injury – which may account for their observed efficacy in reducing CIN.^[11–13]^ Third, by stimulating erythropoietin production via renal hypoxia pathways, they increase hematocrit and enhance oxygen delivery, offering protection during acute ischemic events.^[14,15]^ Fourth, they help restore tubuloglomerular feedback, which reduces glomerular hyperfiltration and intraglomerular pressure, particularly in patients with diabetes or CKD.^[10,16]^ Finally, by increasing ketone body availability, SGLT2 inhibitors enhance myocardial energy efficiency, providing a more oxygen-sparing substrate that may reduce the incidence of HF and mortality.^[17,18]^ Such effects may delay CKD progression, reduce cardiovascular events, improve patients’ quality of life, and decrease healthcare costs, thus alleviating the socioeconomic burden.^[19]^

Although the beneficial effects of SGLT2 inhibitors in treating cardiovascular and renal diseases, including HF and CKD, have been well-documented,^[20]^ their benefits in AMI patients, particularly when administered before PCI, remain inadequately explored due to the absence of large-scale studies and meta-analyses. Small-scale clinical observational studies and randomized controlled trials suggest that SGLT2 inhibitors may improve cardiorenal outcomes, but the conclusions remain controversial, and the clinical efficacy is still uncertain. Major cardiovascular outcome trials (EMPA-REG OUTCOME,^[19]^ DECLARE-TIMI 58,^[21]^ CANVAS^[22]^ and CREDENCE^[23]^) have established the cardiorenal benefits of SGLT2 inhibitors in T2DM patients. However, these agents induce an initial, reversible glomerular filtration rate reduction,^[24]^ prompting current guidelines to contraindicate their initiation during acute illness, including AMI, due to acute kidney injury (AKI) risk.^[25]^ Therefore, this study aims to systematically review and conduct a meta-analysis of the existing literature to evaluate the impact of preoperative SGLT2 inhibitors use on cardiorenal outcomes in AMI patients and explore its potential for improving prognosis.

## 2. Methods

### 2.1. Literature search

Following the Preferred Reporting Items for Systematic Reviews and Meta-Analyses (PRISMA) (2020)^[26]^ guidelines, we conducted a comprehensive search across 3 major databases – PubMed, Web of science, and the Cochrane Library – until December 12, 2024. Both Medical Subject Headings (MeSH) and free-text keywords were used with no language restrictions. The search strategy combined MeSH terms and text words, including: “Sodium-Glucose Transporter 2 Inhibitors” [Mesh], “Acute Kidney Injury” [Mesh], “Percutaneous Coronary Intervention” [Mesh], and “Diabetes Mellitus, Type 2” [Mesh], as well as their synonyms and related terms. To ensure thorough coverage, supplementary methods were employed, such as manually screening reference lists from included articles, examining gray literature sources, and reviewing major cardiovascular conference proceedings from 2020 to 2024. The PubMed search strategy, developed with the assistance of a medical librarian and pretested for sensitivity, utilized Boolean operators and field-specific syntax, as detailed below (Table 1).

**Table 1:** Strategies used for searching electronic databases.

Group	Search terms
#1	(SGLT2i[Title/Abstract]) OR (Sodium Glucose Co-transporter 2 inhibitor[Title/Abstract]) OR (Dapagliflozin[Title/Abstract]) OR (Empagliflozin[Title/Abstract]) OR (Canagliflozin[Title/Abstract]) OR (ertugliflozin[Title/Abstract])
#2	(acute kidney injury[MeSH Major Topic]) OR (Acute Kidney Injuries[Title/Abstract]) OR (Kidney Injuries, Acute[Title/Abstract]) OR (Kidney Injury, Acute[Title/Abstract]) OR (Acute Renal Injury[Title/Abstract]) OR (Acute Renal Injuries[Title/Abstract]) OR (Renal Injuries, Acute[Title/Abstract]) OR (Renal Injury, Acute[Title/Abstract]) OR (Kidney Failure, Acute[Title/Abstract]) OR (Acute Kidney Failures[Title/Abstract]) OR (Kidney Failures, Acute[Title/Abstract]) OR (Acute Kidney Failure[Title/Abstract]) OR (Acute Renal Failure[Title/Abstract]) OR (Acute Renal Failures[Title/Abstract]) OR (Renal Failures, Acute[Title/Abstract]) OR (Renal Failure, Acute[Title/Abstract]) OR (Renal Insufficiency, Acute[Title/Abstract]) OR (Acute Renal Insufficiencies[Title/Abstract]) OR (Renal Insufficiencies, Acute[Title/Abstract]) OR (Acute Kidney Insufficiency[Title/Abstract]) OR (Acute Renal Insufficiency[Title/Abstract]) OR (Kidney Insufficiency, Acute[Title/Abstract]) OR (Acute Kidney Insufficiencies[Title/Abstract]) OR (Kidney Insufficiencies, Acute[Title/Abstract]) OR (contrast-induced nephropathy[Title/Abstract])
#3	(percutaneous coronary intervention[MeSH Major Topic]) OR (PCI[Title/Abstract]) OR (Coronary Intervention, Percutaneous[Title/Abstract]) OR (Coronary Interventions, Percutaneous[Title/Abstract]) OR (Intervention, Percutaneous Coronary[Title/Abstract]) OR (Interventions, Percutaneous Coronary[Title/Abstract]) OR (Percutaneous Coronary Interventions[Title/Abstract])OR (Percutaneous Coronary Revascularization[Title/Abstract]) OR (Coronary Revascularization, Percutaneous[Title/Abstract]) OR (Coronary Revascularizations, Percutaneous[Title/Abstract]) OR (Percutaneous Coronary Revascularizations[Title/Abstract]) OR (Revascularization, Percutaneous Coronary[Title/Abstract]) OR(Revascularizations, Percutaneous Coronary[Title/Abstract])
#4	Diabetes Mellitus, Type 2 [MeSH Major Topic]
#5	#1 AND #2 AND #3 AND #4

### 2.2. Study selection

#### 2.2.1. Inclusion criteria

Confirmed diagnosis of AMI according to the ESC/ACC universal definition^[27]^; Adult population (age ≥ 18 years); and PCI performed during the index hospitalization.

#### 2.2.2. Exclusion criteria

End-stage renal disease: estimated glomerular filtration rate < 15 mL/min/1.73 m^2^ or dialysis dependence; Insufficient serum creatinine measurements (baseline or 48–72 hours post-PCI).

#### 2.2.3. Literature screening and data extraction

Two trained investigators independently performed title/abstract screening and full-text evaluation using Covidence systematic review software. Any discrepancies were resolved through consensus discussion or third-party arbitration by a senior cardiologist.

### 2.3. Risk of bias assessment

All included observational studies were independently evaluated by 2 investigators using the Newcastle–Ottawa Scale (NOS), a validated tool for assessing the methodological quality of non-randomized studies in meta-analyses. The NOS assesses 3 domains: selection of study participants (up to 4 points), group comparability (up to 2 points), and outcome assessment (up to 3 points), with a maximum total score of 9. Discrepancies were resolved through discussion or consultation with a third reviewer when necessary. Studies scoring 7 to 9 were deemed high quality, whereas those scoring 5 to 6 were considered moderate quality.

### 2.4. Population, intervention, comparison, and outcomes framework

Population: AMI patients undergoing PCI (age ≥ 18); Intervention: Pre-PCI SGLT2 inhibitors (any type/dose); Comparator: Standard care without SGLT2 inhibitors; Outcomes: Primary: Incidence of CIN (serum creatinine Δ ≥ 0.5 mg/dL/25% within 72 hours post-contrast); Secondary: Mortality, major adverse cardiovascular events (MACE), Recurrent myocardial infarction, HF readmission.

### 2.5. Statistical analysis

Meta-analysis was conducted using Review Manager 5.3 software. The odds ratio (OR) with 95% confidence intervals (CI) was used as the effect measure. Heterogeneity among the included studies was assessed using the chi-square test and I² statistic. A fixed-effects model was applied if no statistical heterogeneity was detected; otherwise, a random-effects model was used.

### 2.6. Data extraction

Data extraction included the following: first author’s name, publication year, country, participant characteristics, sample size (the SGLT2 inhibitors group and the control group), follow-up duration, and outcome measures. Data extraction was independently performed by 2 authors to ensure accuracy.

### 2.7. Ethical considerations

This study utilized data from publicly accessible databases without involving direct human participation; thus, ethical approval and informed consent were not applicable.

## 3. Results

### 3.1. Study selection

**Figure 1.**
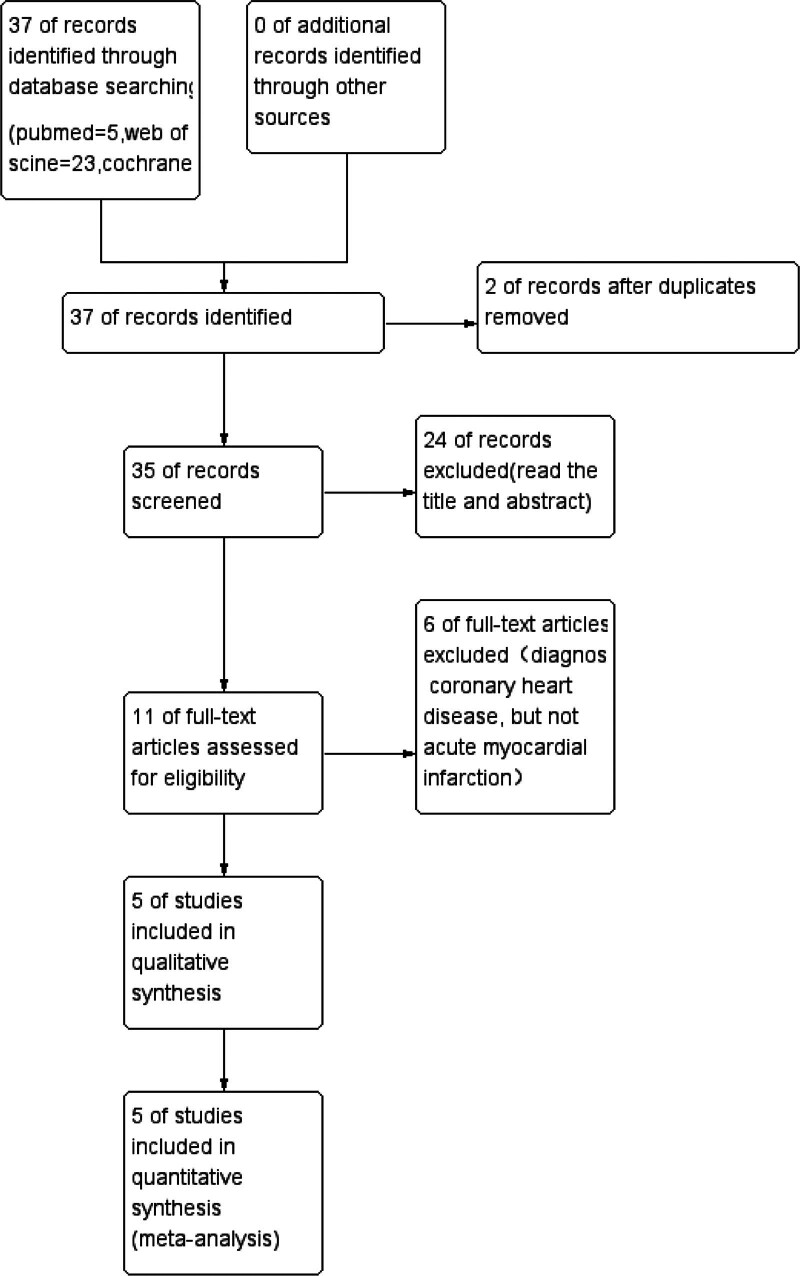
Flow diagram of the meta-analysis.

The systematic literature search identified 37 potentially relevant citations. After removing duplicates using NoteExpress V4.0 and performing manual verification, 35 unique records remained for screening. Through a thorough two-phase selection process, which included title/abstract screening followed by full-text evaluation, 5 case-control studies met our predefined inclusion criteria.^[28–32]^ The PRISMA-compliant flow diagram (Fig. 1) provides a detailed outline of the study selection process, including the reasons for exclusion at each stage.

### 3.2. Characteristics of included studies:

This systematic review included 5 case-control studies,^[28–32]^ which collectively comprised 3301 patients (SGLT2 inhibitors group: n = 665; control group: n = 2636) from various geographic regions. The studies were published between 2023 and 2024, with sample sizes ranging from 312 to 1024 participants per study. The key study characteristics, including population demographics, inclusion/exclusion criteria, SGLT2 inhibitors treatment protocols, and outcome assessment methods, are summarized in Table 2.

**Table 2:** Basic characteristics of included studies.

Author (year)	Location	Diagnosis	Sample size		Intervention		Follow-up time (mo)	Outcomes
			SGLT2i group	Control group	SGLT2i group	Control group		
Cai et al^[28]^	CHN	AMI	278	1561	Dapagliflozin	Blank control	24	*,†
Kültürsay et al^[29]^	Turkey	STEMI	130	163	Dapagliflozin/Empagliflozin	Blank control	6	*,†
Paolisso et al^[31]^	Italy	AMI	83	314	Unmentioned	Blank control	24	*,†,‡,§
Paolisso et al^[30]^	Italy	AMI	111	535	Unmentioned	Blank control	24	*,†,§,‖
Chen et al^[32]^	CHN	STEMI	63	63	Canagliflozin	Blank control	3	*,†,‡,‖

AMI = acute myocardial infarction, STEMI = ST-segment elevation myocardial infarction.

* CIN = contrast-induced nephropathy.

† Mortality.

‡ MACE = major adverse cardiovascular events.

§ Heart failure re-hospitalization.

‖ Re-infarction.

### 3.3. Risk of bias assessment

The quality assessment revealed that all 5 studies included in this meta-analysis were of moderate to high quality. Chen et al^[32]^ and Cai et al^[28]^ obtained full NOS scores (9/9), Paolisso et al^[30]^ and Paolisso et al^[31]^ scored 8, and Kültürsay et al^[29]^ scored 7. All studies achieved full marks in the “Selection” domain, indicating robust participant selection and representativeness. Score variations were primarily in the “Outcome Assessment” domain, likely due to differences in follow-up duration and outcome definitions. Overall, the high methodological quality enhances the internal validity of this meta-analysis and minimizes the risk of major bias. However, since all the studies were retrospective, causal inferences remain limited, highlighting the need for future prospective studies to confirm these findings. The results of the risk of bias assessment are presented in Table 3.

**Table 3:** Newcastle–Ottawa scale (NOS) ratings of included studies.

Study	Selection (max 4 stars)	Comparability (max 2 stars)	Outcome (max 3 stars)	Total stars (out of 9)
Chen et al^[32]^	4	2	3	9
Kültürsay et al^[29]^	3	2	2	7
Paolisso et al^[30]^	4	2	2	8
Paolisso et al^[31]^	4	2	2	8
Cai et al^[28]^	4	2	3	9

### 3.4. Outcomes

#### 3.4.1. Primary outcomes

**Figure 2.**
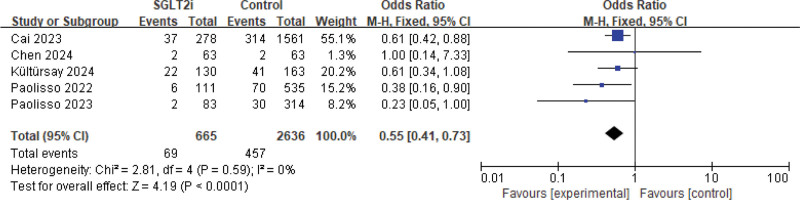
Meta-analysis of CIN between SGLT2 inhibitors and control. CIN = contrast-induced nephropathy, SGLT2 = sodium-glucose cotransporter-2.

The 5 studies that assessed the incidence of CIN in AMI patients who received SGLT2 inhibitors before undergoing PCI are shown in Figure 2. The meta-analysis using a fixed-effects model revealed that preoperative SGLT2 inhibitors administration significantly reduced the incidence of CIN (OR = 0.55, 95% CI: [0.41, 0.73], *P* < .0001), demonstrating a statistically significant difference.

#### 3.4.2. Secondary outcomes

**Figure 3.**
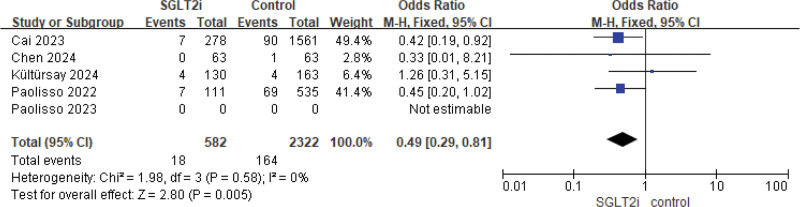
Meta-analysis of mortality.

In terms of in-hospital mortality, 5 studies compared AMI patients undergoing PCI with or without preoperative use of SGLT2 inhibitors (Fig. 3). The fixed-effects model meta-analysis revealed a significant reduction in in-hospital mortality with the preoperative use of SGLT2 inhibitors (OR = 0.49, 95% CI: [0.29, 0.81], *P* = .005).

**Figure 4.**
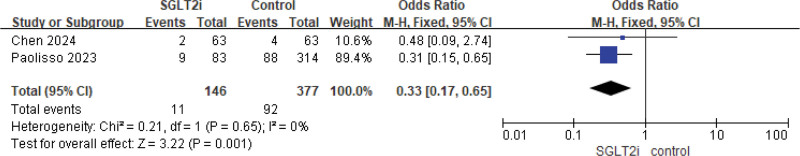
Meta-analysis of MACE. MACE = major adverse cardiovascular events.

Two studies assessed the incidence of MACE during hospitalization in AMI patients who received PCI and compared those with or without preoperative use of SGLT2 inhibitors (Fig. 4). The fixed-effects model meta-analysis demonstrated that preoperative SGLT2 inhibitors significantly reduced the in-hospital MACE rate (OR = 0.33, 95% CI: [0.17, 0.65], *P* = .001).

**Figure 5.**
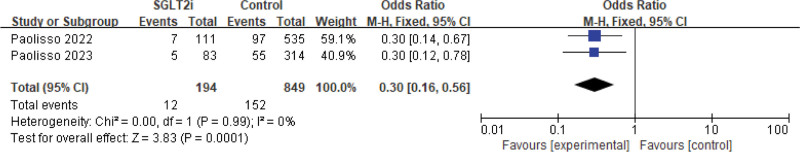
Meta-analysis of re-infarction of the HF hospitalization. HF = heart failure.

Similarly, 2 studies evaluated the rate of HF re-admissions in AMI patients who received PCI with or without preoperative use of SGLT2 inhibitors (Fig. 5). The fixed-effects model meta-analysis revealed that preoperative use of SGLT2 inhibitors significantly reduced HF readmission rates (OR = 0.30, 95% CI: [0.16, 0.56], *P* = .0001), with statistically significant results.

**Figure 6.**
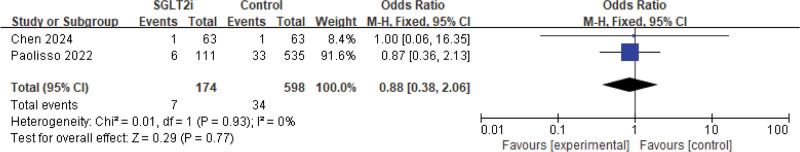
Meta-analysis of recurrent myocardial infarction.

Regarding myocardial re-infarction during hospitalization, 2 studies compared AMI patients who received PCI with or without preoperative use of SGLT2 inhibitors (Fig. 6). The fixed-effects model meta-analysis found no statistically significant difference in myocardial re-infarction rates between the SGLT2 inhibitors and the control group (OR = 0.88, 95% CI: [0.38, 2.06], *P* = .77).

## 4. Discussion

PCI is the standard therapeutic approach for coronary revascularization in clinical practice.^[33]^ This procedure encompasses techniques such as percutaneous coronary balloon angioplasty, coronary stent implantation, coronary atherectomy, coronary thrombus aspiration, and cutting balloon angioplasty. These interventions are designed to restore coronary blood flow, shorten the duration of myocardial ischemia, reduce myocardial injury, decrease infarct size, and improve myocardial perfusion, thereby enhancing patient survival rates.^[34]^ As a result, PCI is considered the first-line treatment for AMI. However, despite its benefits, complications associated with PCI must not be overlooked.

Among these complications, CIN is one of the most common complications during PCI, and its incidence continuously increasing. It has now become the third leading cause of AKI,^[35]^ typically manifesting within 48 hours of exposure to iodinated contrast agents. CIN is associated with factors such as age, baseline renal function, contrast agent usage, absence of coronary re-perfusion, and complex coronary anatomy.^[36]^ The potential pathophysiological mechanisms include hemodynamic changes induced by iodinated contrast agents, renal cell ischemia and hypoxia, tubular toxicity, and oxidative stress damage.^[37–39]^ CIN is characterized by an increase in serum creatinine (Scr) levels, either an absolute increase of 0.5 mg/dL or a relative increase of >25% compared with baseline.^[40]^ It severely affects both the short-term and long-term prognosis of AMI patients.^[41,42]^ Moreover, HF can further impact the prognosis of patients, primarily due to the large infarct size and left ventricular remodeling following AMI.^[43,44]^ Although aggressive treatment strategies may improve patients prognosis to some extent, they occasionally fail to prevent the progression of HF.^[45,46]^ Therefore, reducing complications in patients with AMI, especially the occurrence of CIN, is the key to improving prognosis. It is imperative to explore renal protection strategies before PCI to reduce the incidence of CIN and mortality after PCI in AMI patients, thereby improving their prognosis. Currently, there is no consensus on the prevention and treatment of CIN.^[43]^ Strategies primarily include hydration, reducing contrast agent dosage, and using low-osmolar or iso-osmolar nonionic contrast agents.^[40,44]^ Recently, previous studies have demonstrated that SGLT2 inhibitors have renal protective effects,^[45–48]^ and basic research has also shown that SGLT2 inhibitors can alleviate renal injury in mice with myocardial infarction.^[49]^ As clinical trials progress, the indications for SGLT2 inhibitors are gradually expanding,^[50]^ encompassing T2DM, chronic HF, and CKD.^[51]^ However, some case reports suggest that SGLT2 inhibitors may increase the risk of AKI, possibly due to their diuretic effects, low blood pressure, and reduced blood volume, which can decrease the glomerular filtration rate and lead to AKI. Some researchers even recommend discontinuing SGLT2 inhibitors perioperatively during PCI to reduce the incidence of AKI.^[52]^ Therefore, there is ongoing debate regarding whether SGLT2 inhibitors are beneficial for AMI patients undergoing PCI.

This study employed a meta-analysis approach to systematically evaluate the relationship between pre-PCI use of SGLT inhibitors and the incidence of CIN, mortality, myocardial re-infarction rate, and HF readmission rate in AMI patients. Specifically, SGLT2 inhibitors lowered the risk of CIN by 45% (OR = 0.55, 95% CI: 0.41–0.73, *P* < .0001). They also reduced the risk of death by 51% (OR = 0.49, 95% CI: 0.29–0.81, *P* = .005) and lowered the chance of MACE by 67% (OR = 0.33, 95% CI: 0.17–0.65, *P* = .001). In patients with HF, the use of SGLT2 inhibitors was associated with a 70% decrease in hospital re-admissions (OR = 0.30, 95% CI: 0.16–0.56, *P* = .0001). A network meta-analysis by Yang et al (2022)^[53]^ also revealed similar results, with clear benefits in kidney outcomes, HF hospitalizations, cardiovascular deaths, and overall deaths. A recent meta-analysis by Dimitriadis et al (2024)^[54]^ also revealed that long-term use of SGLT2 inhibitors clearly reduced the risk of CIN in patients with type 2 diabetes undergoing PCI. Our results align with a large meta-analysis by Mascolo et al (2021),^[55]^ which demonstrated that SGLT2 inhibitors significantly lowered the risks of MACE, myocardial infarction, HF, and all-cause mortality in diabetic patients, especially those with high cardiovascular risk. Collectively, these findings show that SGLT2 inhibitors have steady protective effects on the heart and kidneys. They may also provide renal protection during PCI, especially in high-risk patients with diabetes or CKD, and support better clinical management of AMI. The mechanisms of SGLT2 inhibitors may enhance the effects of conventional hydration strategies and facilitate reduced contrast volume usage. Notably, the cardiovascular benefits identified in this analysis align with findings from major trials such as EMPA-REG OUTCOME^[8]^ and DAPA-HF.^[21]^ However, the meta-analysis revealed no significant difference in myocardial re-infarction rates between the groups (RR = 0.88, 95% CI: 0.38–2.06, *P* = .77). In contrast, Wu et al (2022)^[56]^ demonstrated cardioprotective effects of SGLT2 inhibitors in preclinical models, including reduced infarct size and improved myocardial remodeling. The absence of a significant effect in our analysis may stem from limitations such as small sample sizes, retrospective study designs, and heterogeneity in endpoint definitions across studies. Nonetheless, the robust mechanistic basis and growing clinical evidence support the need for large-scale randomized trials to further evaluate the efficacy of SGLT2 inhibitors in reducing reinfarction and improving outcomes, especially in high-risk AMI populations.

Although SGLT2 inhibitors provide proven cardiorenal benefits, their safety in the acute AMI setting warrants attention. Adverse effects such as hypotension and euglycemic diabetic ketoacidosis (euDKA) should be considered before they are broadly applied around the time of PCI. Their natriuretic and diuretic properties, while useful for HF, may worsen volume depletion and lead to hypotension, especially when used with other diuretics or vasodilators.^[51]^ euDKA, although uncommon, has been increasingly reported in acute and perioperative settings. Clinicians should assess these risks cautiously and consider pausing SGLT2 inhibitor therapy during episodes of hemodynamic or metabolic instability. Additional studies are needed to better define their safety in acute coronary settings, especially in patients prone to hypovolemia or ketoacidosis.

Despite its strengths, this meta-analysis has notable limitations. First, all 5 studies were retrospective, introducing potential selection bias and confounding factors, and limiting causal interpretations. Second, the overall sample size was modest, with individual studies enrolling 312 to 1024 patients, which may reduce generalizability and statistical robustness. Third, although statistical heterogeneity for outcomes such as CIN, MACE, mortality, HF readmission, and re-infarction was low (*I*^2^ = 0%), clinical and methodological variability persists, and statistical homogeneity does not eliminate the possibility of clinical or methodological variability. Differences in baseline characteristics (e.g., renal function, glycemic control), endpoint definitions, and SGLT2i administration protocols may contribute to unmeasured heterogeneity. Fourth, formal publication bias assessment (e.g., funnel plots, Egger test) was not performed due to the limited number of studies (n < 10), in line with current guidance. Nonetheless, the consistently favorable trends across studies suggest a low likelihood of significant publication bias, although this cannot be completely excluded. Fifth, a pooled subgroup meta-analysis could not be conducted due to the lack of sufficient subgroup data in the included studies. As a result, it remains unclear whether certain subgroups – such as those with or without diabetes, HF, or CKD – experience differential benefits from SGLT2 inhibitors therapy. Sixth, most of the studies included in this meta-analysis did not specify whether standardized hydration regimens or iso-osmolar contrast agents were used – both of which are well-established factors influencing the risk of CIN. The absence of this information may introduce unmeasured confounding, which should be carefully addressed in future studies evaluating the renoprotective effects of SGLT2 inhibitors.

Clinically, several key questions remain unresolved. These include the ideal timing of SGLT2i initiation in relation to PCI, appropriate dosing in acute settings, and long-term effects on renal outcomes, ventricular remodeling, and HF recurrence. These issues are especially relevant given that SGLT2 inhibitors are typically used chronically in type 2 diabetes patients, with limited data on their safety and efficacy during acute myocardial ischemia. Our findings align with the SGLT2-I AMI PROTECT registry,^[33]^ a large real-world study involving over 2000 diabetic AMI patients undergoing PCI, which reported significant reductions in CI-AKI, in-hospital cardiovascular death, arrhythmias, long-term MACE, and HF hospitalization with chronic SGLT2i use. These observations highlight the need for future prospective, stratified trials based on renal function, diabetes status, and prior SGLT2i exposure. Accordingly, future research should expand upon current findings through large-scale, randomized, multicenter trials to validate the clinical efficacy of SGLT2 inhibitors in AMI patients undergoing PCI. These studies should aim to determine the optimal timing of initiation (e.g., before vs after PCI), define appropriate dosing strategies in acute coronary settings, and evaluate long-term effects on renal function, ventricular remodeling, and recurrent cardiovascular events.

Bridging these evidence gaps will facilitate the development of standardized treatment protocols and support broader clinical adoption across diverse patient populations and healthcare settings. Among these, patients at high bleeding risk (HBR) warrant particular attention due to their complex therapeutic needs and vulnerability to both thrombotic and hemorrhagic complications. In such patients, concerns over bleeding frequently constrain the use or duration of antithrombotic strategies and may deter the administration of nephroprotective interventions because of hemodynamic compromise. The recently validated PRECISE-HBR score^[57]^ – based on factors such as age, hemoglobin, white blood cell count, renal function, and bleeding history – has proven effective in identifying patients at elevated bleeding risk. In this context, SGLT2 inhibitors may offer a therapeutic advantage, as their cardiorenal benefits are not linked to increased bleeding. Given their mechanisms – including volume regulation, endothelial stabilization, and anti-inflammatory action – SGLT2 inhibitors may offer incremental benefits in HBR patients without increasing hemorrhagic risk. Integrating bleeding risk stratification tools such as the PRECISE-HBR score into future randomized trials could better define the safety and efficacy of SGLT2 inhibitors in this population, improve external validity, and guide individualized therapy for vulnerable AMI patients undergoing PCI.

In summary, this meta-analysis indicates preoperative use of SGLT2 inhibitors significantly reduces the risks of CIN, all-cause mortality, MACE, and HF readmission in AMI patients undergoing PCI. These findings support their role in cardiorenal protection and suggest SGLT2 inhibitors as a promising adjunct to standard preventive strategies. Nevertheless, potential risks – such as hypotension and euDKA – require careful consideration in the acute phase. Future large-scale, randomized, prospective studies are warranted to validate the efficacy, determine the optimal timing, and identify high-risk subgroups most likely to benefit, enabling safe and rational clinical application.

## Acknowledgments

We sincerely thank the staff of the Departments of Cardiology and Oncology at Rongchang District People’s Hospital for their support during the preparation of this manuscript. We also gratefully acknowledge the constructive feedback provided by the peer reviewers and editorial team, which significantly contributed to improving the quality of the article.

## Author contributions

**Conceptualization**: Yue Li.

**Data curation**: Wanyao Zhang.

**Formal analysis**: Yue Li.

**Funding acquisition**: Rui Li.

**Methodology**: Yue Li.

**Resources**: Yue Li.

**Software**: Yue Li.

**Supervision**: Rui Li.

**Validation**: Rui Li.

**Writing – original draft**: Yue Li.

**Writing – review & editing**: Rui Li .
